# Adapting international clinical trials during COVID-19 and beyond

**DOI:** 10.1177/17407745231154215

**Published:** 2023-02-11

**Authors:** Kamala Thriemer, Tamiru Shibiru Degaga, Mohammad Shafiul Alam, Bipin Adhikari, Rupam Tripura, Mohammad Sharif Hossain, Michael Christian, Najia K Ghanchi, Hellen Mnjala, Sophie Weston, Benedikt Ley, Angela Rumaseb, Dagimawie Tadesse, Tedla Teferi, Daniel Yilma, Grant Lee, Holger Unger, Inge Sutanto, Ayodhia Pitaloka Pasaribu, Prakash Ghimire, Mohammad Asim Beg, Ric N Price

**Affiliations:** 1 Global and Tropical Health Division, Menzies School of Health Research and Charles Darwin University, Darwin, NT, Australia; 2 College of Medicine & Health Sciences, Arba Minch University, Arba Minch, Ethiopia; 3 International Centre for Diarrhoeal Disease Research, Bangladesh (ICDDR, B), Dhaka, Bangladesh; 4 Mahidol-Oxford Tropical Medicine Research Unit, Faculty of Tropical Medicine, Mahidol University, Bangkok, Thailand; 5 Centre for Tropical Medicine and Global Health, Nuffield Department of Clinical Medicine, University of Oxford, Oxford, UK; 6 Eijkman-Oxford Clinical Research Unit, Jakarta, Indonesia; 7 Department of Pathology and Microbiology, Aga Khan University, Karachi, Pakistan; 8 Arba Minch General Hospital, Arba Minch, Ethiopia; 9 Jimma University, Jimma, Ethiopia; 10 Department of Parasitology, Faculty of Medicine, University of Indonesia, Jakarta, Indonesia; 11 Department of Child Health, Universitas Sumatera Utara, Medan, Indonesia; 12 Central Department of Microbiology, Tribhuvan University, Kirtipur, Nepal

**Keywords:** COVID-19, clinical trial management, malaria trials, virtual training, remote monitoring, clinical trials network

## Abstract

**Background:**

The COVID-19 pandemic and resulting restrictions, particularly travel restrictions, have had significant impact on the conduct of global clinical trials. Our clinical trials programme, which relied on in-person visits for training, monitoring and capacity building across nine low- and middle-income countries, had to adapt to those unprecedented operational challenges. We report the adaptation of our working model with a focus on the operational areas of training, monitoring and cross-site collaboration.

**The new working model:**

Adaptations include changing training strategies from in-person site visits with three or four team members to a multi-pronged virtual approach, with generic online training for good clinical practice, the development of a library of study-specific training videos, and interactive virtual training sessions, including practical laboratory-focused training sessions. We also report changes from in-person monitoring to remote monitoring as well as the development of a more localized network of clinical trial monitors to support hybrid models with in-person and remote monitoring depending on identified risks at each site. We established a virtual network across different trial and study sites with the objective to further build capacity for good clinical practice–compliant antimalarial trials and foster cross-country and cross-study site collaboration.

**Conclusion:**

The forced adaptation of these new strategies has come with advantages that we did not envisage initially. This includes improved, more frequent engagement through the established network with opportunities for increased south-to-south support and a substantially reduced carbon footprint and budget savings. Our new approach is challenging for study sites with limited prior experience but this can be overcome with hybrid models. Capacity building for laboratory-based work remains difficult using a virtual environment. The changes to our working model are likely to last, even after the end of the pandemic, providing a more sustainable and equitable approach to our research.

## Background

The World Health Organization declared the COVID-19 outbreak a pandemic on 11 March 2020.^1^ In response, many countries implemented strict restrictions including physical distancing, nationwide lockdowns, working from home directives and interstate and overseas travel bans. These restrictions had a significant impact on the conduct of clinical trials globally, resulting in difficulties recruiting patients into ongoing studies and halting the start of new trials.^2^ In the United States alone, thousands of trials were suspended in the first year of the pandemic,^3,4^ and more than half of all planned trials were not initiated.^5^ Research efforts were diverted to tackle pandemic-related issues, such as the United Kingdom National Health Service issuing guidance to pause new or ongoing studies which weren’t prioritized COVID-19 studies.^6^ Other regulatory agencies and funding bodies provided guidance on conducting trials during the pandemic, taking into account patients and staff safety measures.^7,8^ In addition to safety concerns for patients and trial staff, these restrictions led to unprecedented operational challenges for everyday management of clinical trials.

Our clinical research programme is primarily focused on optimizing the treatment and prevention of malaria. At the start of the COVID-19 pandemic, one large multicentre trial was underway with sites in Bangladesh, Indonesia and Ethiopia (NCT 03916003), another trial had begun in Nepal (NCT 04079621) and further trials were in preparation in Cambodia, Indonesia, Ethiopia, Pakistan (NCT 04411836) and Papua New Guinea (NCT 05426434). All those studies were planned under the assumption that study sites will be easily accessible for training purposes, study site initiation and monitoring and continuous capacity building.

The introduction of pandemic-related travel restrictions in early 2020 rendered physical visits to study sites by international investigators and study teams impossible. These unprecedented operational challenges forced us to revise our working model significantly. Here, we discuss adaptations to our working model, with a focus on operational areas of training, monitoring and cross-site collaboration and discuss the implications for the future.

## The new working model

### Training

Most trials in resource-limited settings rely on study-specific training and face-to-face site initiation visits to ensure quality of patient care and build capacity. In many instances, this requires coordinating teams travelling from a central hub (in our case, northern Australia) to each of the study sites for 5–8-day visits. Teams usually include a study coordinator (to provide protocol-specific training to the field team), a laboratory expert (to cover aspects around diagnostics and sample processing) and a data manager.

While the opportunities to use technology to reduce travel and support training were available before the COVID-19 pandemic, they weren’t used. Following stringent international travel restrictions implemented by the Australian Government, our team was forced to consider new approaches to deliver training and site initiation visits. We adopted a multi-pronged approach: (1) generic online training for good clinical practice (GCP); (2) study-specific training videos and (3) interactive virtual training sessions (Table 1).

**Table 1.** Old and new working model.

	Old working model	New working model
Training	Face to face at study site by coordinating team members (often up to three team members such as study coordinator, laboratory expert and data manager)	Online training materials for teams to watch in their own time Interactive virtual sessions for protocol training Practical virtual training with laboratory staff
Monitoring	On-site study monitoring by Australian-based trial monitor	Virtual central monitoring Network of local study monitors to conduct on-site visits Hybrid approaches depending on identified risks (risk-based approaches)
Ongoing informal communication	Very limited, mostly with PIs at conferences	Chatgroups with study teams and coordinating team for real-time clarification

PI: principal investigator.

As previously, study protocol and Standard Operating Procedures were made available to all study staff and the site teams were encouraged to familiarize themselves with the respective documents prior to any other training. Generic resources available through the Global Health Network^9^ on GCP were used. A library of more than 30 training videos was developed with generic as well as study-specific content. These were uploaded as open access to YouTube^10^ for team members to watch in their own time prior to interactive training sessions. Study teams were then invited to attend interactive virtual training sessions through Zoom to discuss the information covered in the training material. These virtual sessions focused on patient screening and informed consent, study procedures at enrolment and during follow-up and safety assessments, including management of adverse events. Data entry and management were covered within the same sessions. Case studies and scenario discussions were used to highlight specific issues that might be encountered. When necessary the content was translated in real time into the local language by site investigators. Complimentary sessions covered practical training with expert laboratory staff to ensure adequate use of point-of-care diagnostics and sample processing. In these practical sessions, trainers asked the teams to install several mobile phone cameras connected to the same Zoom session to observe sample handling and processing from different angles.

Since the beginning of the pandemic, we have conducted more than 20 training sessions across three studies, six study teams, with more than 60 participants and in average three to four trainers from the central hub. Approximately half of those trainings included hands-on sessions.

### Monitoring

Adopting risk-based monitoring strategies has been slow, despite good evidence that this can provide an efficient approach to study monitoring.^11^ An important element of risk-based monitoring is off-site or remote monitoring.^12^ In view of the extensive travel restrictions for our Australian coordinating team, most monitoring visits within the last 3 years were undertaken remotely and virtually embracing a risk-based monitoring approach (Table 1). In practical terms, this means that paper-based case record forms, which are source documents for most variables in our studies, were scanned and uploaded onto a secure password-protected server, and data cross-checked and validated for consistency by the off-site study monitor. All other paper-based trial documents (such as site delegation log, training logs, temperature logs, drug accountability and so on), were scanned and uploaded to the same server for virtual monitoring. Informed consent forms were either checked during Zoom calls with the site team or uploaded into a separate secure server to protect highly confidential data.

Traditional on-site monitoring includes observation of procedures in real time. This is a valuable way of ensuring good study conduct. We found it extremely difficult, if not impossible, to replace this element with virtual monitoring. To address this, we quickly built a network of study monitors, who are based closer to the study sites and were less affected by travel restrictions. This required additional virtual one-on-one training and study orientation for those monitors, but ultimately, we envisage a hybrid approach with some on-site monitoring and virtual monitoring visits, depending on identified risks at each site.

### Supporting ongoing communication for quality improvement

Multimedia online chat groups are favoured by many collaborators, but were not used systematically pre-COVID. For most groups, WhatsApp was the most common software and study site–specific groups were established including the on-site study team and the coordinating team members. Those were supplemented in some instances by Facebook messenger groups. These proved to be highly effective and popular means of communication for maintaining contact, real-time clarification of study processes and issues as they arose. Throughout the study period, this allowed all relevant stakeholders to remain informed about the study progress on an informal, yet informative basis. These groups were also used to link queries with more formal monitoring that resulted in quality improvement and ongoing training and support (Table 1).

### Establishing a network for the future

As we moved international engagement to a more virtual environment, progress meetings were either organized by trial with all study sites joining or with individual study sites to discuss site-specific issues. We identified opportunities to bring together study teams from different sites and different trials into a virtual network, with the objective to further build capacity for GCP compliant antimalarial trials and foster cross-country and cross-study-site collaborations.

Since the inception of our trial network, monthly seminars have been held to support sustained capacity building and peer-to-peer support. Until now the focus of these seminars varied from general to study-specific issues. General issues include safety reporting, community (study participant) engagement and patient retention, practical challenges (e.g. setting up trials in remote locations) and sample management and processing. Study-specific topics include the practical challenges of procedures to identify warning signs for haemolysis in trials assessing primaquine and tafenoquine radical cure of patients with *Plasmodium vivax* malaria.^13^ In the future, this forum will be used to share study results, discuss new research priorities and co-develop grant applications and new study protocols.

### Advantages and limitations of the new working model

Since February 2020, there has been a significant shift to virtual engagement using videoconferencing tools that has increased and, in many ways, improved our overall engagement and communication with partners. Previously, most of our interactions occurred during on-site visits, or through phone calls, emails or during conferences and other face-to-face meetings. This approach facilitated strategic planning, but there was often relatively little interaction between these visits. The use of digital technology has become routine, with a significant increase in the frequency of communication within- and between-study teams, leading to more efficient support structures and an important increase in connectivity.

The shift from in-person meetings to videoconferencing has increased equality within an otherwise relatively hierarchical research structure.^14^ Larger groups are now able to share experiences, and this has led to an increased access to scientific discussion for researchers from low- and middle-income countries, for whom costs of travelling are often prohibitive. Our newly established clinical trials network brings together diverse partners to discuss common challenges and share experiences and learnings. Rather than information flowing from an Australian coordination hub to each of the study sites, the online meetings have fostered knowledge sharing between researchers from different study sites fostering cross-fertilization and south-to-south support.

Pre-COVID-19 travel by academics was substantially higher than the average population, and this contributed to a significant carbon footprint.^15–17^ Previously, training and monitoring visits for our clinical research programme (excluding conferences and other meetings) amounted to approximately 96,000 miles travelled per year, to support field sites in Ethiopia, Indonesia, Nepal, Cambodia and Bangladesh. This equates to nearly 12,000 kg of CO_2_ and a cost of approximately US$60,000. Throughout the COVID-19 pandemic, our strategy of virtual engagement has successfully ensured study quality and integrity and made it indefensible to go back to our previous travel schedule, both from an environmental as well as a budgetary perspective.

Despite the clear benefits of online engagement, there are a number of important limitations. First, Internet bandwidth limitations for some of our collaborators render consistent high-quality connectivity difficult, thereby frustrating interactions. Second, knowledge transfer and capacity building for clinical trial conduct using online tools is significantly easier with partners having previous trial experience compared to partners with less clinical trials experience, highlighting the need to use hybrid models. We have developed such an approach with partners in Nepal, where we have adopted a mix of online support and face-to-face support by network members from Bangladesh. Third, hybrid models are perceived to require more time for preparation to ensure similar outcomes. While this is true, this needs to be balanced with time savings from intensive travel commitments. Furthermore, with increasing experience, less preparation is required. Finally, capacity building for laboratory-based work can be extremely difficult using virtual support alone. Despite the use of video-recordings and interactive sessions, this cannot fully replace face-to-face at the bench learning and will require hands-on training.

## Conclusion

The COVID-19 pandemic has led to unprecedented challenges for conducting clinical trials, particularly for large multi-country trials in resource-limited settings. We have successfully adapted our working models to embrace virtual training and remote monitoring options and this has greatly facilitated a strong international network, with advantages that we did not initially envisage. Our new working model is likely to remain our preferred way forward providing a more sustainable and equitable way of delivering clinical trials in our areas of research.

## References

[1] Cucinotta D Vanelli M. WHO declares COVID-19 a pandemic. Acta Biomed 2020; 91: 157–160. 32191675 10.23750/abm.v91i1.9397 PMC7569573

[2] Sathian B Asim M Banerjee I , et al. Impact of COVID-19 on clinical trials and clinical research: a systematic review. Nepal J Epidemiol 2020; 10(3): 878–887. 33042591 10.3126/nje.v10i3.31622 PMC7538012

[3] Asaad M Habibullah NK Butler CE. The impact of COVID-19 on clinical trials. Ann Surg 2020; 272: e222–e223. 10.1097/SLA.0000000000004113 PMC7467053 32541234

[4] Van Dorn A . COVID-19 and readjusting clinical trials. Lancet 2020; 396: 523–524. 32828180 10.1016/S0140-6736(20)31787-6 PMC7440868

[5] Unger JM Xiao H. The COVID-19 pandemic and new clinical trial activations. Trials 2021; 22: 260. 33832534 10.1186/s13063-021-05219-3 PMC8027975

[6] National Institute for Health and Care Research (NIHR). DHSC issues guidance on the impact of COVID-19 on research funded or supported by NIHR, 2020, https://www.nihr.ac.uk/news/dhsc-issues-guidance-on-the-impact-on-covid-19-on-research-funded-or-supported-by-nihr/24469 (accessed 2022).

[7] European Medicines Agency (EMA). Guidance on the management of clinical trials during the COVID-19 (Coronavirus) pandemic, https://ec.europa.eu/health/latest-updates/updated-document-guidance-management-clinical-trials-during-covid-19-coronavirus-pandemic-2022-02-10_en (accessed 2022).

[8] National Health and Medical Research Council (NHMRC). COVID-19: guidance on clinical trials for institutions, HRECs, researchers and sponsors, 2020, https://www1.health.gov.au/internet/main/publishing.nsf/Content/EE207D978A44E4B8CA257FA90081B212/$File/COVID-19%20Guidance%20on%20clinical%20trials%20for%20institutions,%20HRECs,%20researchers%20and%20sponsors.pdf (accessed 2022).

[9] The Global Health Network. Global health trials, https://globalhealthtrials.tghn.org/elearning/ (accessed 2022).

[10] Weston S. Menzies malaria, 2020, https://www.youtube.com/channel/UCb0OgzUvNzsywranSs4PF3g/playlists

[11] Barnes B Stansbury N Brown D , et al. Risk-based monitoring in clinical trials: past, present, and future. Ther Innov Regul Sci 2021; 55(4): 899–906. 33914298 10.1007/s43441-021-00295-8 PMC8082746

[12] ACRO. Establishing risk-based monitoring within a quality-based system as ‘best practice’ for clinical studies, 2019, https://www.acrohealth.org/wp-content/uploads/2019/10/ACRO-RBM-White-Paper-October-2019.pdf

[13] Menzies. Tackling malaria: we are focused on better understanding the deadly parasites that cause human malaria: resources, https://www.menzies.edu.au/page/Research/Global_and_Tropical_Health/Malaria/ (accessed 2022).

[14] Dobson DA Wolberg AS. COVID-19 pandemic perspectives: a scientific silver lining? Res Pract Thromb Haemost 2020; 4(7): 1083–1086. 33134774 10.1002/rth2.12432 PMC7590274

[15] Buchs M. University sector must tackle air travel emissions. The Conversation 2019, https://theconversation.com/university-sector-must-tackle-air-travel-emissions-118929

[16] Nathans J Sterling P. How scientists can reduce their carbon footprint. eLife 2016; 5: e15928. 10.7554/eLife.15928 PMC4829415 27029962

[17] Quinton JN. Cutting the carbon cost of academic travel. Nat Rev Earth Environ 2020; 1: 13.

